# A Social, Not a Natural Science: Engaging With Broader Fields in Health Policy Analysis: Comment on "Modelling the Health Policy Process: One Size Fits All or Horses for Courses?"

**DOI:** 10.34172/ijhpm.2023.8101

**Published:** 2023-06-25

**Authors:** Justin Parkhurst

**Affiliations:** Department of Health Policy, London School of Economics and Political Science, London, UK

**Keywords:** Health Policy, Public Policy, Health Policy Processes, Theories, Frameworks, Models

## Abstract

Powell and Mannion’s recent editorial discusses how different ‘models’ of the policy process have been applied within the health policy field. They present two ways forward for scholarship: more ‘home grown’ development of health-specific models, or deeper engagement with broader public policy scholarship. In this paper I argue for the latter approach for several reasons. First, health policy analysis is a social, not a natural science – and as such is not exceptional to other forms of policy scholarship. Second, many ‘health policy models’ are often grounded in conceptual work from elsewhere (or may not be health specific). Finally, there has been significant work to develop more nuanced understandings of theories, models, and frameworks available to particular analytical tasks and questions. As such, the growing body of global health policy scholarship may find it can benefit more from deeper engagement with existing conceptual work than constructing its own new models in most cases.

 In Powell and Mannion’s^1^ recent editorial: ‘Modelling the Health Policy Process: One Size Fits All or Horses for Courses’ the authors discuss ways that different ‘models’ of the policy process have been applied within the health policy research sector. The authors note that much of the explicit consideration of policy processes and theories by health scholars can be traced back nearly 30 years to the foundational paper of Walt and Gilson^2^ that presents policy analysis as a way to move beyond simple descriptions of health policy content. Powell and Mannion then discuss some more recent reviews of health policy literature, presenting a summary box and table comparing what are termed ‘Policy Process Models’ compared to ‘Health Policy Process Models.’ From this overview, the authors present two options for a way forward in the field: more ‘home grown’ efforts to develop models specific to health, or deeper engagement with the broader public policy scholarship.

 In this comment, I argue for the latter of these approaches. First is because making an argument for ‘home grown’ efforts requires constructing a difficult case for health policy exceptionalism that appears unjustified. The field of public policy (as well as some other related fields) have spent decades specifically considering questions of public policy change, providing a very large corpus of knowledge and theory that most health scholars are yet to engage with significantly. Additionally, even those models seen in health examples (labelled ‘Health Policy Process Models’) often draw on theories and concepts from elsewhere and are not usually specific to health. Thus, rather than looking to create new approaches tailored to health, a more useful approach to the field may be more critical thinking about the tasks and goals of the analysis being done, in order to harness relevant concepts more effectively in the future.

 While there are no doubt some political features of the health sector which appear fairly specific — such as the importance of clinicians epistemic power, or the oligopolistic influence of pharmaceutical companies — it is difficult to argue that health policy-making is inherently different from other social policy sectors. Health policy is (principally) made by governments (often at national level, but at times decentralised — eg, state governments in the United States or India). It is usually held to be redistributive (but at times captured by elites). It is shaped by actors who work through networks; who source and exert power in different ways; who actively (re)construct problems and potential solutions; and who work through existing institutional arrangements in pursuit of goals and interests. The forms of power and concentration of power may be more likely to take particular forms compared to, say, education, environment, or criminal justice — but overall these are common features of public policy making which have significant conceptual and empirical literature on which to draw.^3,4-8^ And the conceptual development around these ideas has dated back half a century or more, with some classic examples drawing on health as well as other public policy cases — without holding health to be somehow fundamentally unique. One example of this is John Kingdon’s *Agendas, Alternatives, and Public Policy* — famous for its establishment of the ‘3 streams’ model of policy change — but based on research conducted in the 1970s looking at both health and transportation policies in the United States.^9^ As such, health policy research must be understood as *policy *research, and not *medical* research — as the subjects of study are policy-making systems, components, and processes — not human anatomy or biochemistry. It is fundamentally a social, rather than a natural science — and the presence of health as the policy concern does not somehow change this. There is thus a real risk that if health policy scholars only look at health (or medical) based sources, they can miss foundational material of relevance necessary to best understand the issue (imagine doing research on lateral epicondylitis treatment, and deciding that only information from the tennis-related literature is relevant).

 A second point, however, is that even examples of ‘home grown’ models may not be that unique to health. Walt and Gilson developed their ‘policy triangle’ in 1994^2^ to highlight the importance of existing policy analysis ideas that can move discussions beyond descriptions of policy content. They state their triangle “…*is a highly simplified model of an extremely complex set of interrelationships*” (p. 355); noting a range of other concepts that are relevant to the components within it. Other ‘Health Policy Process’ models noted by Powell and Mannion further appear to be classified as ‘health’ models because they have been applied to health cases, but not necessarily because they are developed for health specific issues. ‘Networks,’ ‘stages,’ or top-town and bottom-up implementation (‘Multiple implementation theory’) are all examples of this. Similarly, the ‘3I’s’ of ideas, interests, and institutions exist across sectors — pointing users to other developed bodies of theory: ideational, institutional, or interest based.

 The existing reviews mentioned by Powell and Mannion usually find only a limited amount of health policy scholarship that has explicitly engaged with public policy concepts in depth. It therefore seems premature to argue the health policy sector needs greater development of its own ideas. Rather, it may be that the health policy field is increasingly ready to consider and apply existing concepts with greater sophistication and critical engagement.

 One starting point to doing this can be to unpack the different concepts often grouped together as ‘Models.’ In an early edition of *Theories of the Policy Process*, for instance, chapters by Ostrom^10^ and Schlager^11^ try to do just this — by distinguishing between ‘frameworks,’ ‘theories,’ and ‘models’ in the policy sciences based on the tasks they undertake — organising inquiry, understanding relationships between elements, or making precise assumptions. In this light, the ‘horses for courses’ terminology of Powell and Mannion’s article appears particularly apt. While some past reviews of health policy literature have tried to count whether or not a health case study has used a policy concept — it is arguably more important to consider what are the appropriate tools for specific analytical tasks.

 In their 2007 review of health policy literature, Gilson and Raphaely note that there is great scope to move beyond descriptions of ‘what happened’ to apply conceptual insights and explore ‘what explains what happened.’^12^ But they further identify a set of criteria by which to judge qualitative work attempting to do this. The first criterion listed is: “clarity of research question and appropriateness of design to question”^12^ (p. 300). ‘Horses for courses,’ as such, can be taken to mean explicit consideration of the empirical questions being asked, with considering the most useful analytical tools to answer them.

 As research questions are specified and unpacked, scholars in the health policy will no doubt find they have a very large number of tools to draw upon. Understanding ‘what explains what happened’ usually requires more specific questions such as: why particular issues are prioritised; how problems are constructed; whose interests are represented; how implementation proceeded; how systems facilitated or hindered particular choices, etc.

 Theories, models and frameworks from the policy sciences exist to help understand each of these. Yet it is also worth noting that the most commonly cited work in this field has developed from scholarship in the United States and Europe. Today, as championed by Walt and Gilson, much more work in the health policy space looks at health reforms and policy-making in lower income settings. As such it may be important to reflect on policy concepts in combination with conceptual insights derived from broader political analyses in these settings as well. Examples include early work on post-colonial politics and political economy,^13-15^ as well as more recent regional applications of politics and public policy approaches (eg, in Latin American or African settings^16,17^).

 The number of scholars applying public policy concepts to health issues is expanding dramatically. And, indeed, there may now be several policy concerns around which health research have engaged more deeply than some other policy fields — providing useful lessons for other public policy scholarship. One example of this is the growing work on the commercial determinants of health and political role of corporations whose products that can cause public harms (eg, tobacco and alcohol industries^18-20^). Yet (health) policy-making remains a social and political process — not a uniquely medical one. In the past, health examples have been used alongside other social policy case studies for foundational work in public policy scholarship. They can continue to do so, but there is still a wealth of untapped potential to apply existing concepts and approaches to the expanding field of global health policy scholarship.

## Ethical issues

 Not applicable.

## Competing interests

 Author declares that he has no competing interests.
